# Prognosis after cardiac arrest

**DOI:** 10.1186/cc9721

**Published:** 2011-03-11

**Authors:** O Touma, G Hadjipavlou

**Affiliations:** 1 John Radcliffe Hospital, Oxford, UK

## Introduction

Unconscious, mechanically ventilated survivors of cardiac arrest account for a large number of intensive care admissions. Such patients have a spectrum of outcomes, ranging from brain death to good recovery. Predicting the final neurological outcome during the early post-resuscitation phase is required and has been the centre of multiple studies.

## Methods

We performed a literature review of studies assessing outcome predictors following cardiac arrest. We also reviewed national and international guidelines on the subject.

## Results

In comatose adult patients after cardiac arrest, and who have not been treated with hypothermia and who do not have confounding factors, the absence of the pupillary light response and corneal reflex at day 3 provides the most reliable predictor of poor outcome. The absence of vestibulo-ocular reflexes at ≥24 hours and a GCS motor score of 2 or less at ≥72 hours are less reliable. The presence of myoclonus is not recommended for predicting poor outcome. The presence of myoclonic status epilepticus on day 1 is strongly associated with poor outcome. Several EEG findings are strongly, but not invariably associated with a poor outcome. Malignant EEG findings are associated with false predictive rate of 3%. Bilateral absence of the N_2_O cortical response to median nerve stimulation during somatosensory evoked potentials (SSEP) predicts poor outcome after 24 hours of cardiac arrest with FPR of 0.7%. There are no high-level studies that support the use of any imaging modality to predict outcome. There is some evidence that loss of distinction between grey and white matter on CT scan predicts poor outcome. Several studies have confirmed a relationship between serum neuron-specific enolase and poor outcome but the cut-off points are not clear. The value of serum S1000 and cerebrospinal fluid creatine kinase brain isoenzyme measurement is very limited. Therapeutic hypothermia after cardiac arrest complicates prognostication and evidence evaluating predictors of poor outcome in this situation is limited.

## Conclusions

Reliable predictors of poor outcome after cardiac arrest are the absence of the pupillary and corneal reflexes at day 3. Bilateral absence of the N_2_O cortical response to median nerve stimulation during SSEP at day 1 is highly accurate. The use of EEG, CT, and neurological biomarkers is not reliable. Limited studies are available for predicting outcome after therapeutic hypothermia.
